# Supplementary material for: Meter-scale heterostructure printing for high-toughness fiber electrodes in intelligent digital apparel
Source: Nat Commun. 2025 May 9;16:4320. doi: 10.1038/s41467-025-59703-4 (PMC12064786; doi:10.1038/s41467-025-59703-4)
Supplement: Supplementary file 1 — Supplementary Information [file 41467_2025_59703_MOESM1_ESM.pdf]

Supplementary Information for Nature Communications

Meter-Scale Heterostructure Printing for High-Toughness Fiber Electrodes in Intelligent Digital Apparel

Gun-Hee Lee[1,2,†], Yunheum Lee[3,†], Hyeonyeob Seo[3,†], Kyunghyun Jo[4], Jinwook Yeo[5], Semin Kim[6], Jae-Young Bae[7], Chul Kim[3], Carmel Majidi[8], Jiheong Kang[9], Seung-Kyun Kang[7], Seunghwa Ryu[5], and Seongjun Park[1,10,11,12,13]*

[1] Medical Research Center, Seoul National University, 103 Daehak-ro, Jongno-gu, Seoul 03080, Republic of Korea

[2] Departments of Cogno-Mechatronics Engineering and Optics & Mechatronics Engineering, Pusan National University, Busan 46241, Republic of Korea

[3] Department of Bio and Brain Engineering, Korea Advanced Institute of Science and Technology (KAIST), 291 Daehak-ro, Yuseong-gu, Daejeon 34141, Republic of Korea

[4] Program of Brain and Cognitive Engineering, Korea Advanced Institute of Science and Technology (KAIST), 291 Daehak-ro, Yuseong-gu, Daejeon 34141, Republic of Korea

[5]Department of Mechanical Engineering, Korea Advanced Institute of Science and Technology (KAIST), 291 Daehak-ro, Yuseong-gu, Daejeon 34141, Republic of Korea

[6] Graduate School of Semiconductor Technology, Korea Advanced Institute of Science and Technology (KAIST), 291 Daehak-ro, Yuseong-gu, Daejeon 34141, Republic of Korea

[7] Department of Materials Science and Engineering, Seoul National University, 1 Gwanak-ro, Gwanak-gu, Seoul 08826, Republic of Korea

[8] Department of Mechanical Engineering, Carnegie Mellon University, Pittsburgh, PA, 15213 USA

[9] Department of Chemistry, Seoul National University, Seoul, 1 Gwanak-ro, Guwanak-gu, Seoul 08826, Republic of Korea

[10] School of Transdisciplinary Innovations, Seoul National University, 1 Gwanak-ro, Guwanak-gu, Seoul 08826, Republic of Korea

[11] Department of Biomedical Science, College of Medicine, Seoul National University, 103 Daehak-ro, Jongno-gu, Seoul 03080, Republic of Korea

[12] Interdisciplinary Program in Bioengineering, College of Engineering, Seoul National University, 1 Gwanak-ro, Guwanak-gu, Seoul 08826, Republic of Korea

[13] Department of Transdisciplinary Medicine, College of Medicine, Seoul National University Hospital, 103

Daehak-ro, Jongno-gu, Seoul 03080, Republic of Korea

†These authors contributed equally to this work.

*Corresponding authors: S. P. (seongjunpark@snu.ac.kr)

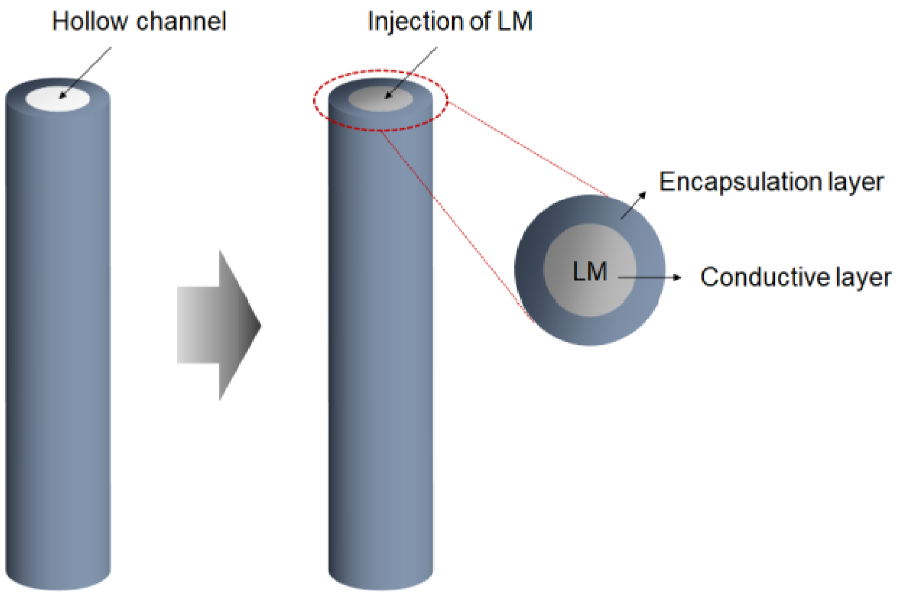

Supplementary Fig. 1| Conventional liquid metal (LM) injection-based stretchable fiber.

The conventional approach for producing LM-based fibers involves injecting liquid metal into the hollow core of a stretchable fiber. However, this method requires an additional encapsulation layer over the conductive layer, making it inadequate for use as an electrode for integration with electronic components. As a result, only a limited range of applications that do not involve direct contact have been demonstrated, such as strain sensors, Joule heaters, and triboelectric textiles, as outlined **supplementary table 1**.

**Ref. number**	**Materials (Hollow fiber)**	**Applications**
[1]	LM/Geniomer	Strain sensor, triboelectric textile
[2]	LMP/PVDF-HFP-TFE+PEGDA	Heater, triboelectric textile, sensor
[3]	LM/SEBS	Triboelectric textile, sensor
[4]	LM/TPU	Heater, sensor
[5]	LM/PFA	Wireless antenna
[6]	LM/TPE-0A/C/CIP	Strain sensor
[7]	LM/SEBS	Triboelectric textile, sensor

Supplementary table 1| Materials and applications about the conventional Liquid metal (LM) -based fibers

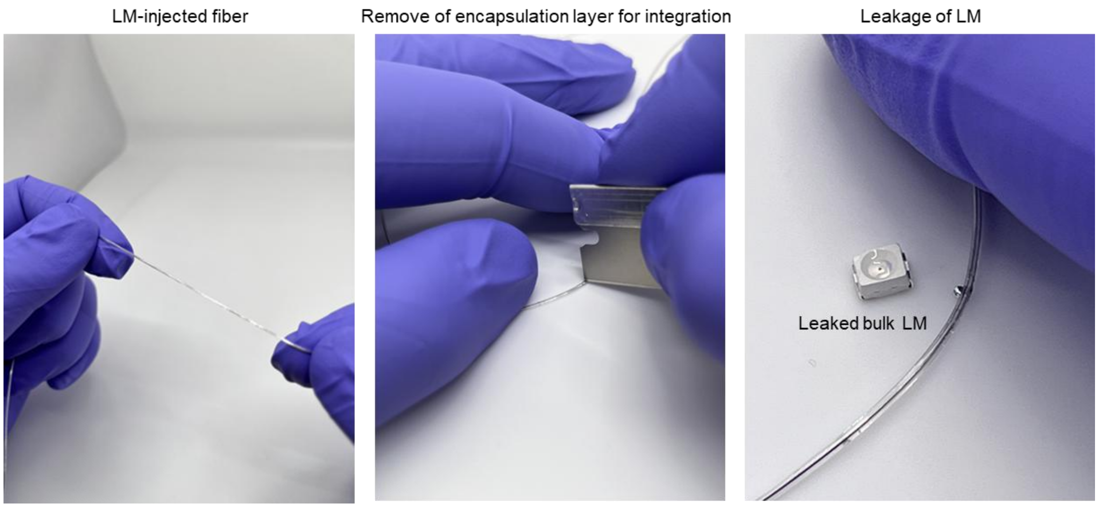

Supplementary Fig. 2| Leakage of LM-injected fiber during integration with electronic chip.

The LM-injected fiber is encapsulated with an elastomer, which must be removed when integrating the fiber with an electronic chip featuring a conductive LM layer. However, the removal of this encapsulation layer can result in bulk LM leakage due to its inherent fluidity, thereby compromising the stability and reliability of integration with solid conventional chips.

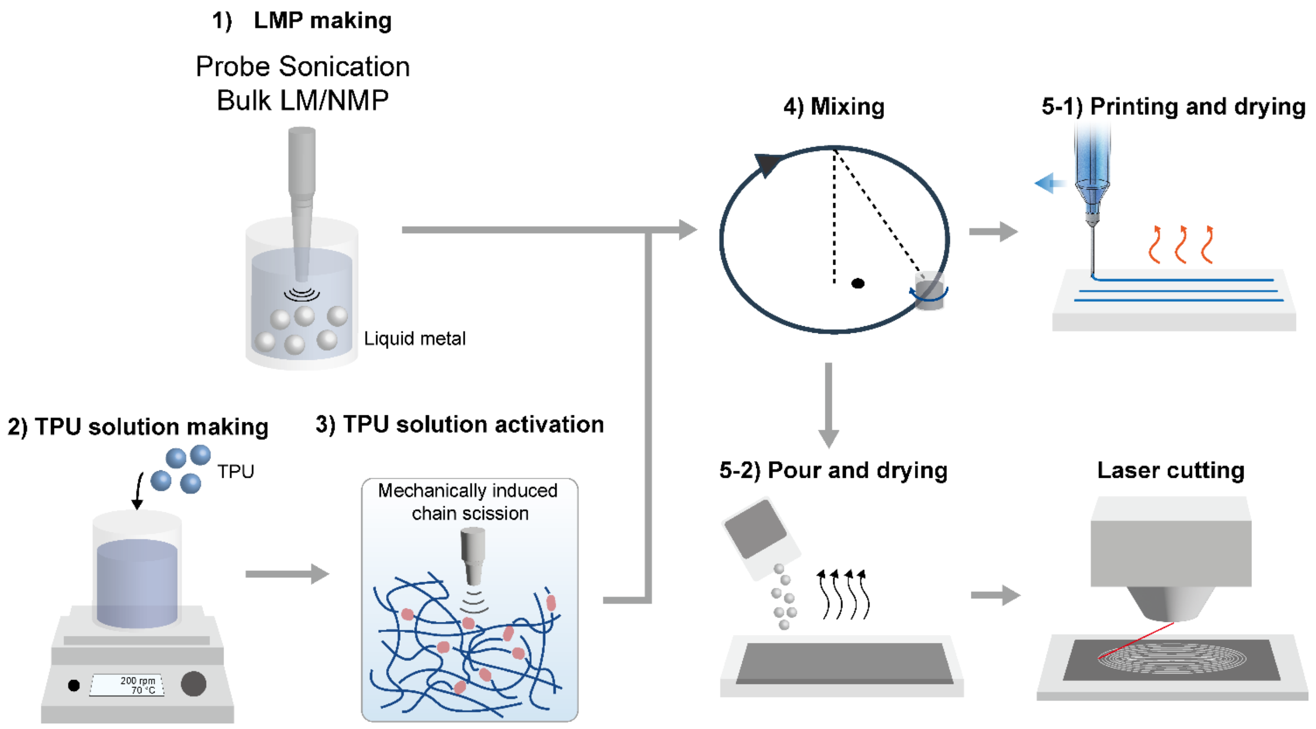

Supplementary Fig. 3| Fabrication methods of TSF.

To create a stable conductor that combines high toughness, stretchability, and conductivity, TSF was designed so that the liquid metal (LM) and polymer phases remain effectively separated while LM particles are stably incorporated into the polymer matrix. This was achieved by activating TPU chains, thereby promoting favorable interactions between LM and TPU. Leveraging these interactions, we successfully fabricated large quantities of fibers via printing and laser cutting.

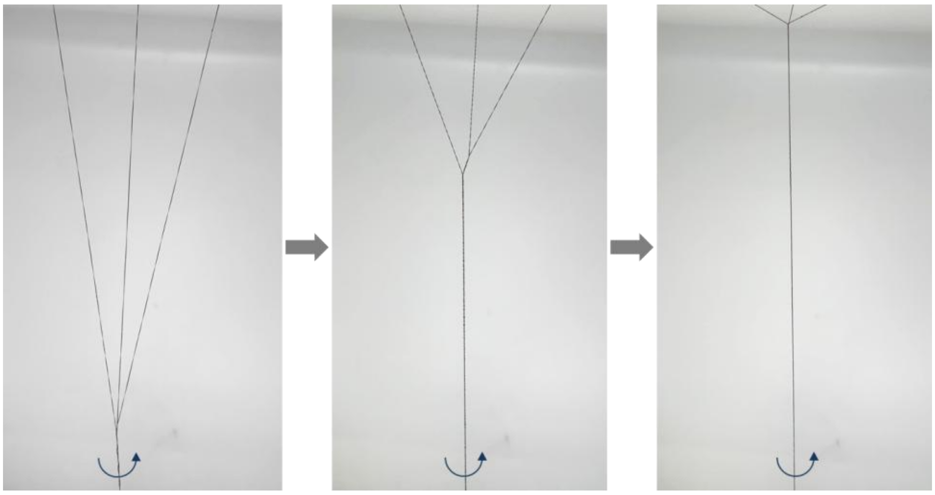

Supplementary Fig. 4| Photograph of twisting TSF.

To enhance the mechanical toughness and electrical stability, three strands of TSF are twisted together to form a TSF^{tw}.

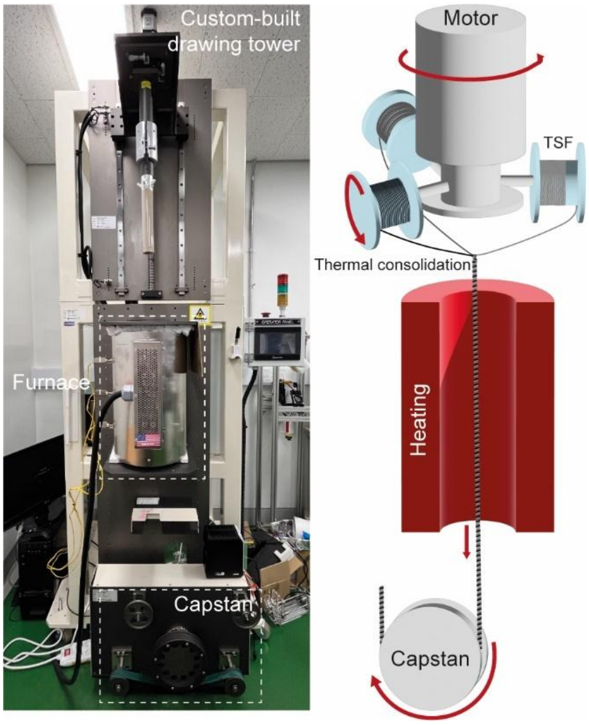

Supplementary Fig. 5| Photograph of twisted TSFs sintering process.

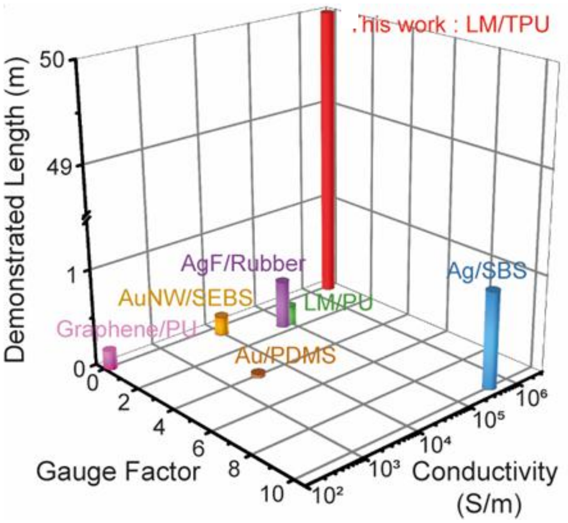

Supplementary Fig. 6| Gauge factor, initial conductivity, and demonstrated length of reported stretchable fiber electrodes.

Our LMP-based TSF exhibits both high initial conductivity, low gauge factor, and long demonstrated length compared to previous referenced research studies. The detailed values are described in **supplementary table 2**.

Ref, number	Materials	Gauge Factor	Initial conductivity(S/m)	Demonstrated length(m)
This work	LM/TPU	0.085	2.22×10^{6}	> 50
[8]	Graphene/PU	0.05	1.24×10^{2}	0.2
[9]	Au/PDMS	4	3.21×10^{3}	0.035
[10]	AuNWs/SEBS	0.5	10^{4}	0.2
[11]	AgF/Rubber	1.5	7.38×10^{4}	0.5
[12]	LM/PU	1.5	10^{5}	0.2
[13]	Ag/SEBS	10	4.4×10^{5}	1

Supplementary table 2| Gauge factor, initial conductivity, and demonstrated length of reported stretchable fiber electrodes.

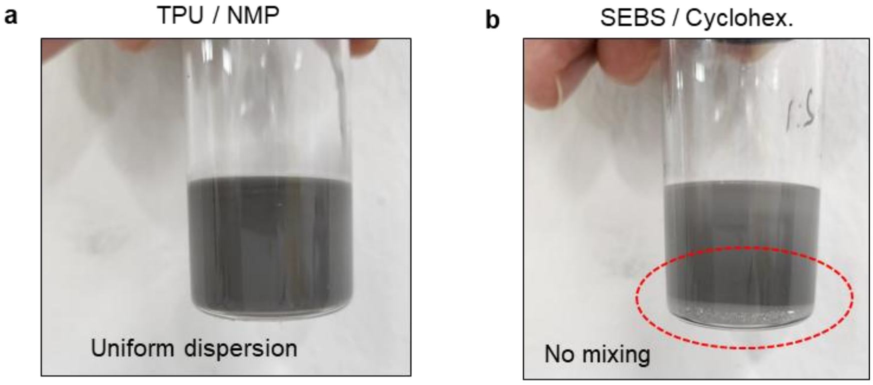

Supplementary Fig. 7| Photograph of LM ink with different polymer. a, LMP dispersed in TPU solution **b,** LMP dispersed in SEBS solution.

Surface gallium oxide readily mixes with polar polymers like TPU. However, when liquid metal is dispersed in non-polar polymers like SEBS, it doesn't disperse as microparticles or mix well in the solution.

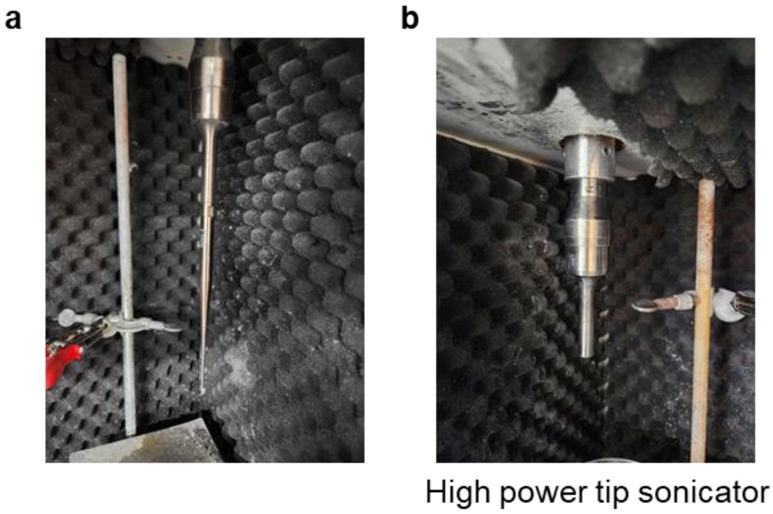

Supplementary Fig. 8| Tip sonicator for formation of LMP ink a, Photograph of sonicator with 2mm tip. **b**, Photograph of sonicator with 25mm tip.

To functionalize the TPU by breaking chemical bonds, a high-power tip sonication process is necessary. Utilizing a 25 mm diameter tip, we can effectively break down the TPU during ink formation.

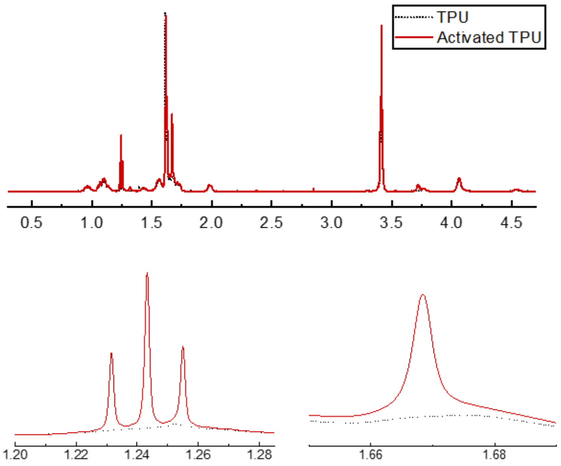

Supplementary Fig. 9| Proton nuclear magnetic resonance (1H NMR) spectrum of TPU before (black) and after tip sonication (red).

Here, the triplet corresponds to the hydrogen peak from the carbon adjacent to the amine group, while the singlet represents the hydrogen peak in the amine group.

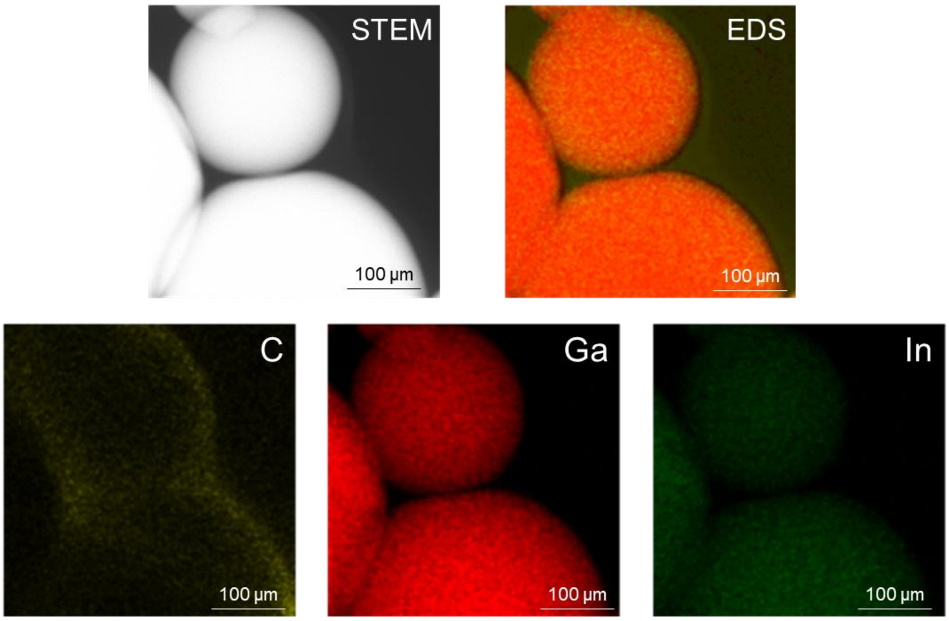

Supplementary Fig. 10| TEM-EDS analysis of bridged TPU on LMPs.

TEM image and corresponding EDS elemental mapping of TPU-LMP ink, showing the distribution of carbon, gallium, and indium. The EDS mapping confirms that TPU (carbon) is bridged to the surface of liquid metal particles (gallium and indium), indicating interfacial connection.

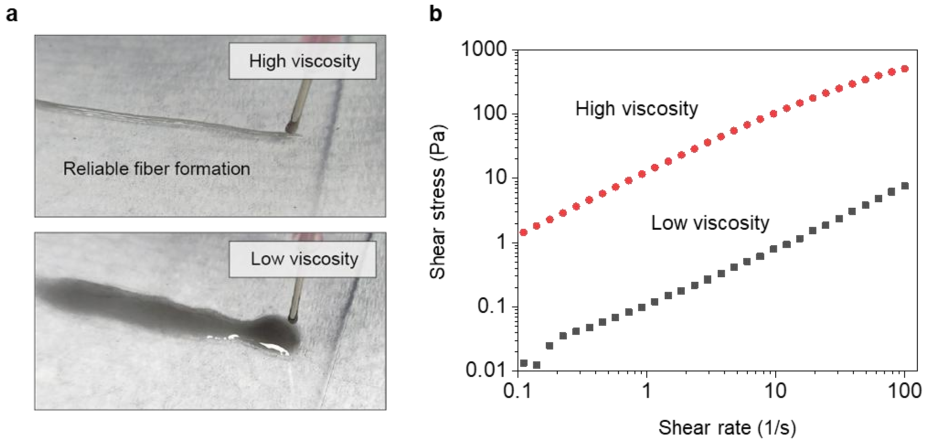

Supplementary Fig. 11| Rheological property of ink. a, Photograph of fiber formation with high and low viscosity solution. **b,** Shear stress according to shear rate of high and low viscosity solution.

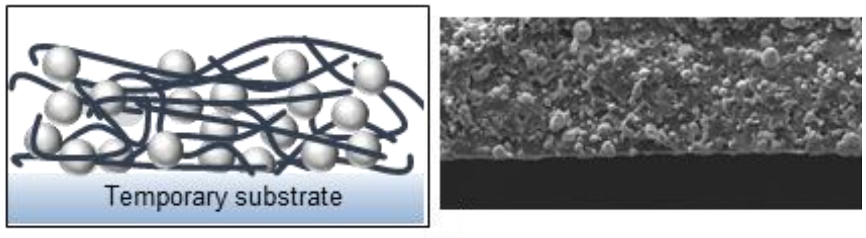

Supplementary Fig. 12| Side view of LMP/bare TPU fiber without phase separation.

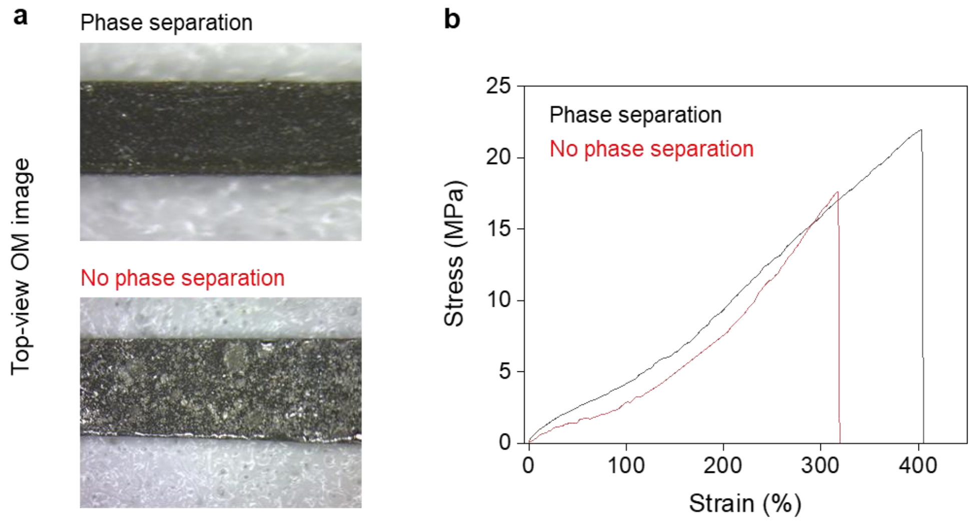

Supplementary Fig. 13| LMP/TPU fiber with and without phase separation. a, Top-view OM image of LMP/TPU fibers with and without phase separation. **b,** Mechanical property of LMP/TPU fibers with and without phase separation.

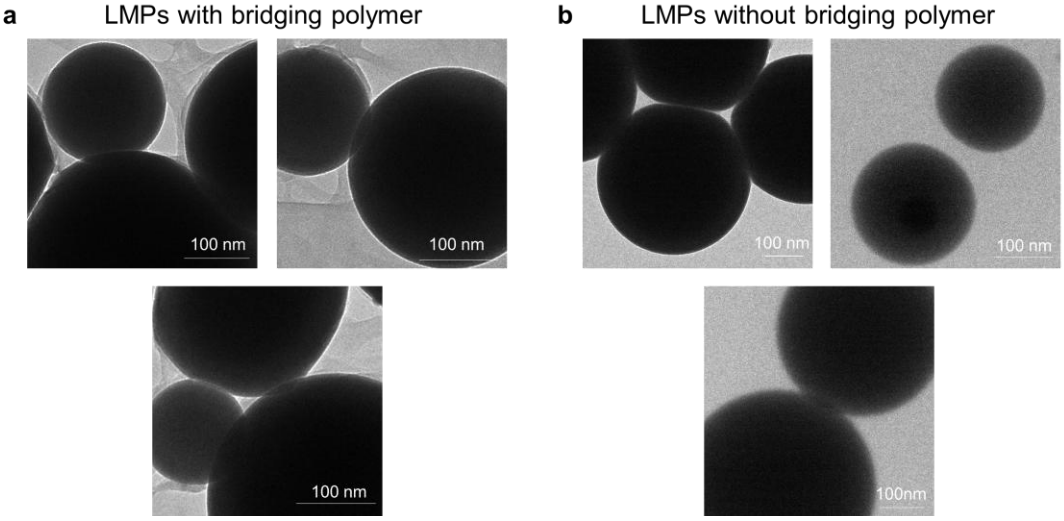

Supplementary Fig. 14| TEM image of LMP. a, LMP with bridging polymer **b,** LMP without bridging polymer.

The amine groups in the functionalized TPU attach to the oxide layer on the surface of the LMP, effectively bridging the particles. Without functionalized TPU, the particles are not bridged and remain dispersed.

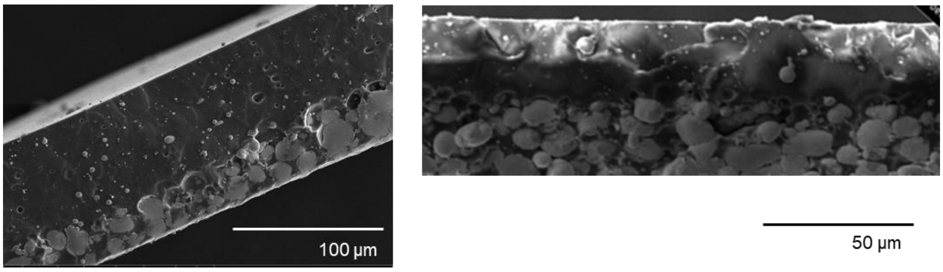

Supplementary Fig. 15| Side-view SEM images of TSF.

The side SEM images of TSF display the dense arrangement of LMP, which creates a conductive pathway within the TPU.

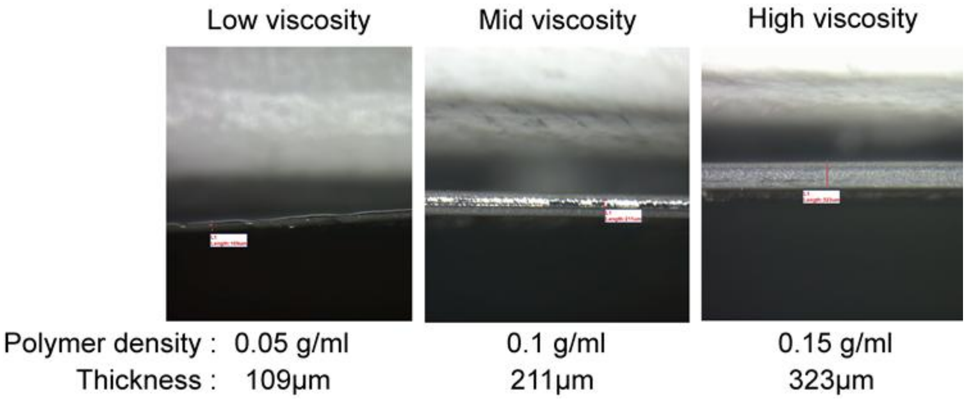

Supplementary Fig. 16| Thickness control of fiber.

By controlling the viscosity of the solution, the thickness of the fiber can be adjusted.

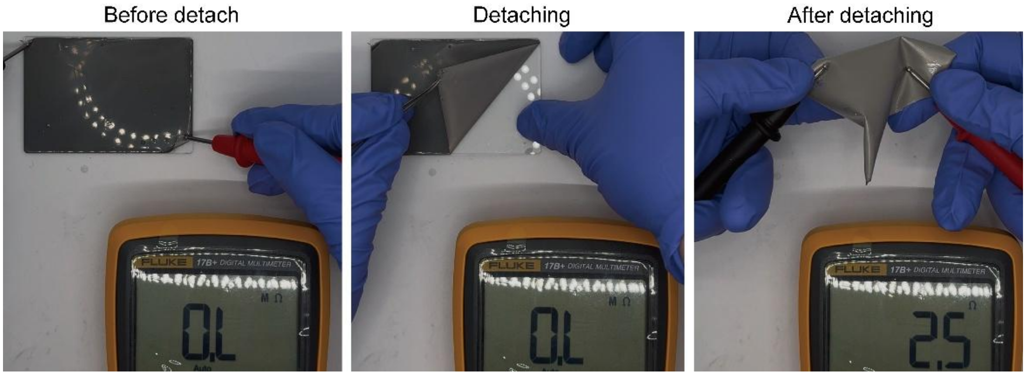

Supplementary Fig. 17| Photograph of the electrical connection of the TSF during the peel-off process.

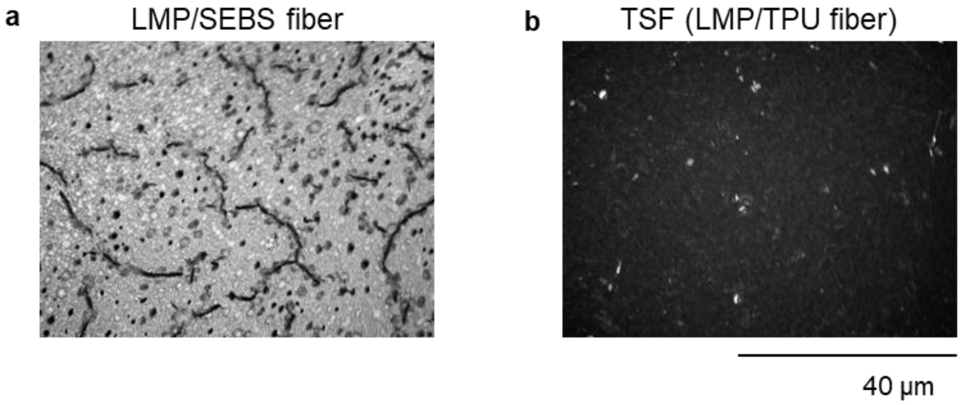

Supplementary Fig. 18| OM images of fibers. a, LMP/SEBS-based fiber **b,** LMP/TPU-based TSF

The solvent used to dissolve SEBS is cyclohexane, a non-polar and highly volatile solvent. Due to its rapid evaporation, it leads to the formation of non-uniform films with voids.This non-uniformity lowers the material's toughness and increases its susceptibility to external stimuli.

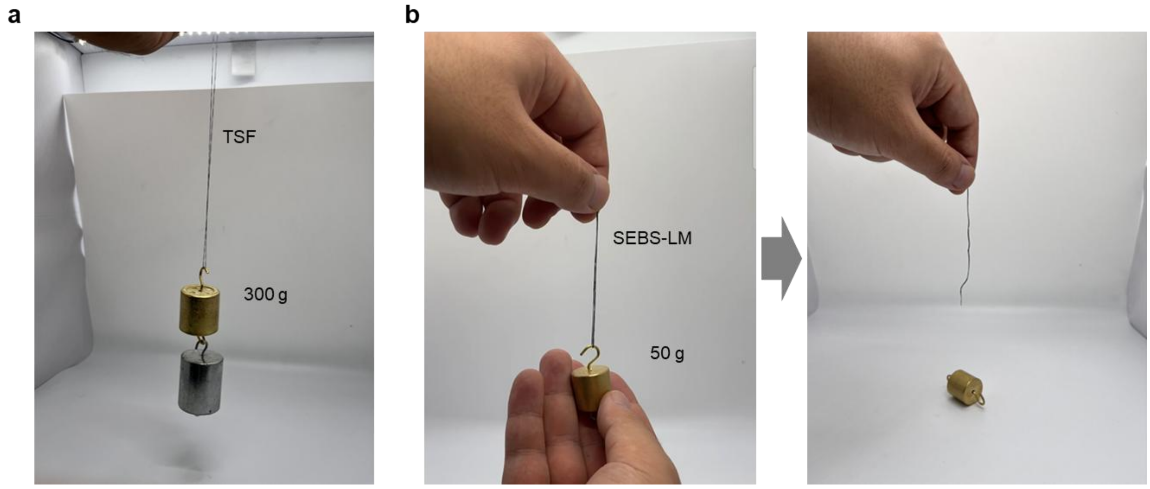

Supplementary Fig. 19| Fiber with low toughness. a, Photograph of TSF connected with 300g weight. **b,** Photograph of SEBS-LM fiber connected with 50g weight. The low toughness of SEBS-LM makes it susceptible to tearing when subjected to weight.

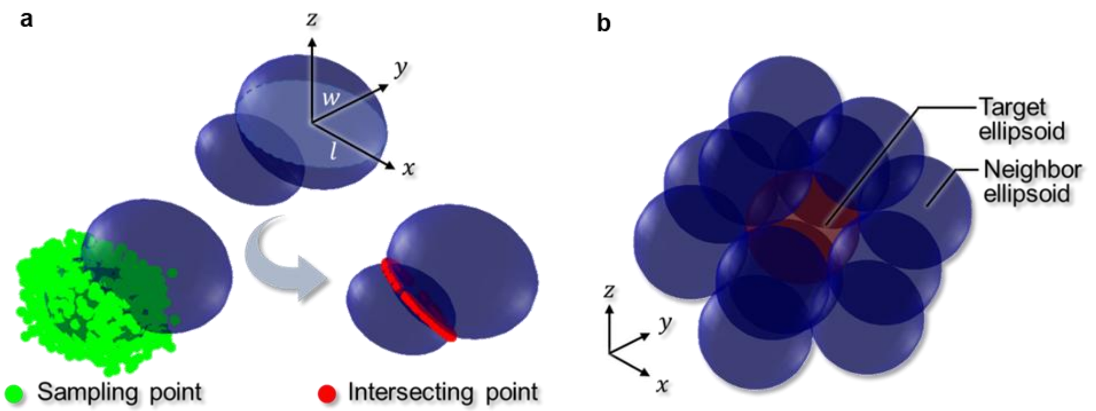

Supplementary Fig. 20| Simulation of Liquid metal composite. a, Schematic illustration of calculating of intersecting area between two ellipsoids. **b,** Arrangement of ellipsoids.

In this research, we conducted a simulation to investigate the deformation of liquid metals and changes in contact area between them under external deformation conditions for a liquid metal-based composite material.

Instead of directly calculating the contact area between the two liquid metals, we opted to calculate the intersection area. This approach was chosen because liquid metals have relatively low stiffness, and it enhances the efficiency of the simulation. Initially, we assumed that the liquid metal would maintain its ellipsoidal shape even when subjected to external deformation. Additionally, we considered the deformation caused by the contact between the two liquid metals to be negligible, allowing them to intersect. When the composite material experienced strain (ε_x^∞ and ε_y^∞=ε_z^∞ represent external strain along the x-axis and y or z-axis, respectively.), the axial length (l) of the liquid metal is theoretically estimated as follows,[14]

$$\frac{l}{2R_0} = 1 + \frac{\frac{5}{1-v^2}\left[(2-v)\varepsilon_x^\infty - (1-2v)\varepsilon_y^\infty\right]}{6 + 15\frac{\gamma}{ER_0}}$$

where R_0, E, v, and γ are initial radius of liquid metal, Young's modulus of matrix, Poisson's ratio of matrix, and surface tension of liquid metal, respectively. Additionally, by considering the conservation of liquid metal volume, the aspect ratio (l/w) is calculated as follows,

$$\left(\frac{l}{w}\right)^{\frac{2}{3}} = 1 + \frac{\frac{5}{1-v^2}\left[(2-v)\varepsilon_x^\infty - (1-2v)\varepsilon_y^\infty\right]}{6 + 15\frac{\gamma}{ER_0}}$$

And we assumed that the center position ($[x_c, y_c, z_c]$) of the liquid metal would be updated based on an affine transformation, expressed as,

$$[x_c, y_c, z_c] = \left[(1+\varepsilon_x^\infty)x_{c0}, \frac{y_{c0}}{\sqrt{(1+\varepsilon_x^\infty)}}, \frac{z_{c0}}{\sqrt{(1+\varepsilon_x^\infty)}}\right]$$

where a subscript of '0' denotes the initial state.

Calculating the intersecting area of two ellipsoids analytically is generally challenging, so we introduced the concept of Monte Carlo method to compute the intersecting area between the two ellipsoids. As shown in Supplementary Fig. 17. a, by generating a point through random sampling and evaluating whether the point is included in the two ellipsoids, the intersecting point set was constructed. With the intersecting point set, the conic equation of ellipse was fitted using the least square method and the area of the ellipse, which is used as intersecting area, was calculated.

For the arrangement of liquid metal within the matrix, we assumed that the liquid metal is stacked in a

hexagonal close-packed (HCP) structure. Consequently, we set up the simulation so that the target ellipsoid and its neighbors are placed in an HCP array and all ellipsoids have the same initial radius as shown in Supplementary Fig. 17. b. We then obtained the major axis length (l), minor axis length (w), averaged intersecting area with neighbor ellipsoids for the target ellipsoid. This process was simulated by incrementing the simulation step strain ($\Delta\varepsilon_x^\infty$) by 10% until reaching a maximum ε_x^∞ was 160%.

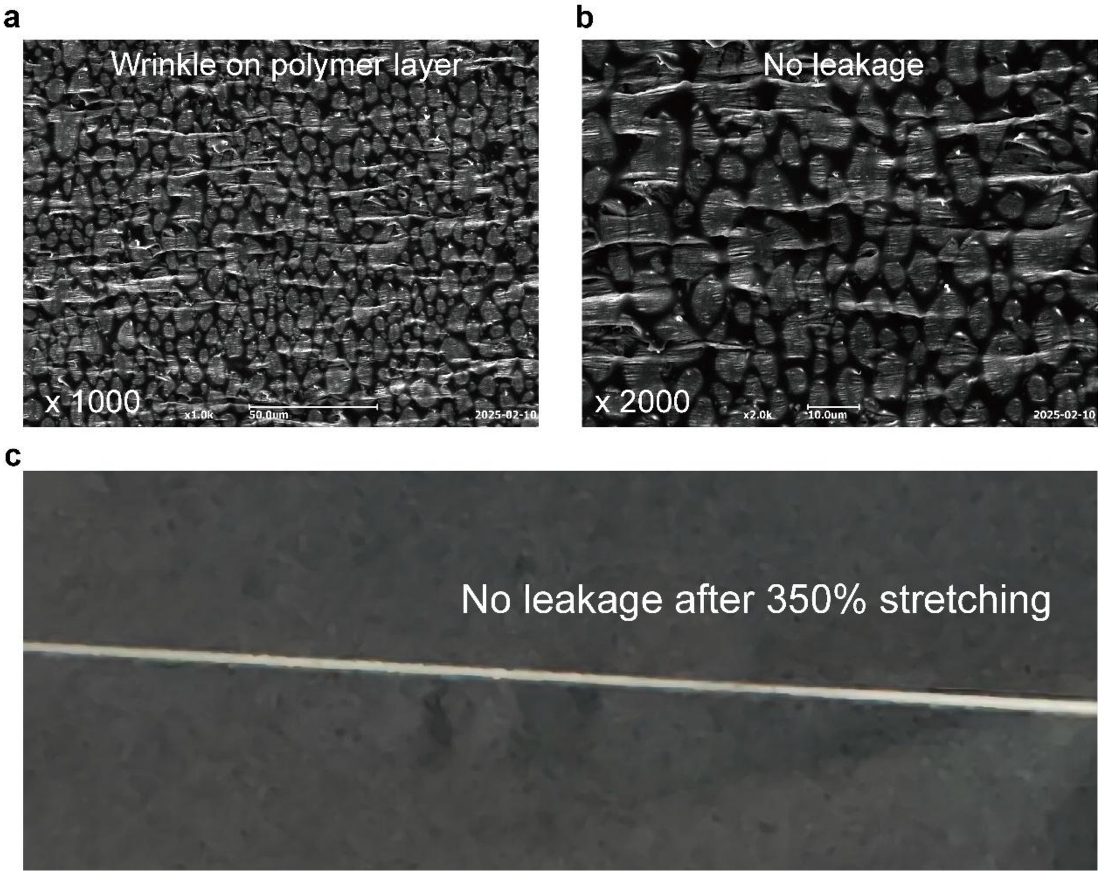

Supplementary Fig. 21| SEM image and photograph of TSF after 350% stretching. a, b SEM image of TSF after stretching**. c,** Photograph of TSF after stretching

SEM image and photograph of TSF after 350% stretching confirm that, even at 350% strain, the polymer encapsulation on the bottom surface allows the structure to remain intact without leakage of the LMP.

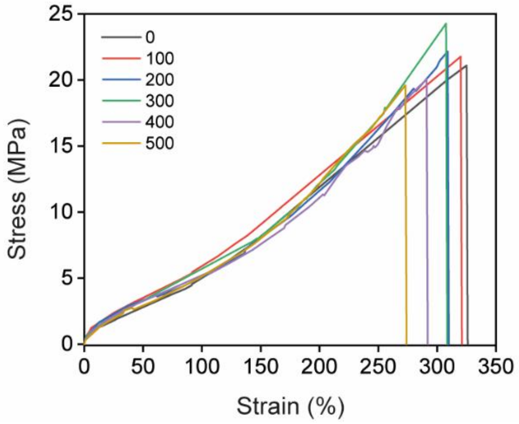

Supplementary Fig. 22| Stress-strain curve of TSF^{tw} according to twisting insertion.

The toughness of TSF^{tw} increases when TI is increased up to its bias angle.

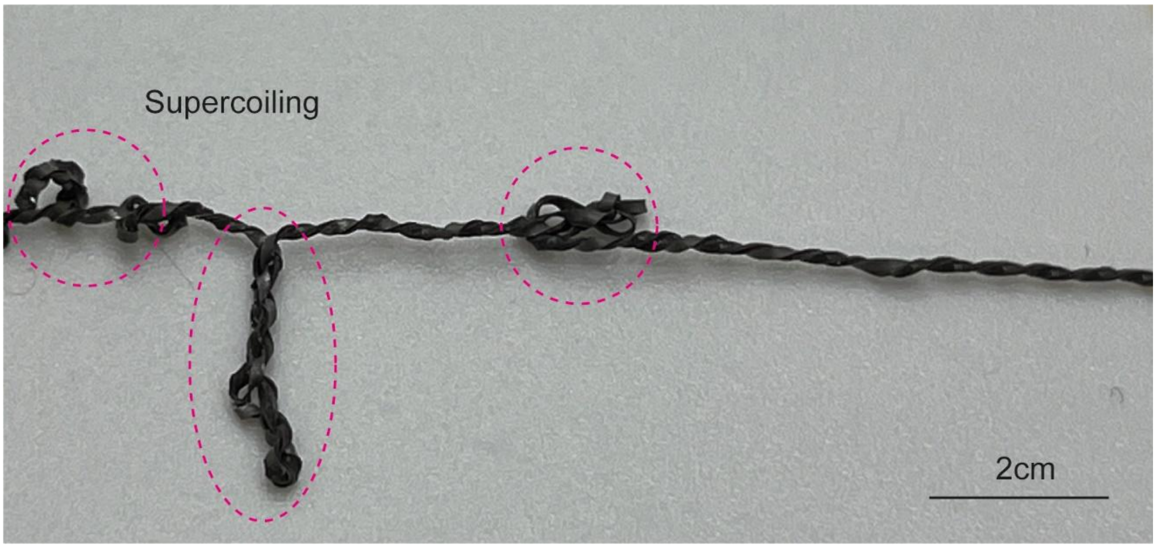

Supplementary Fig. 23| Photograph of supercoiled TSF^{tw}

If the twisting angle of the fiber bundle exceeds its bias angle, supercoiling of fiber is observed.

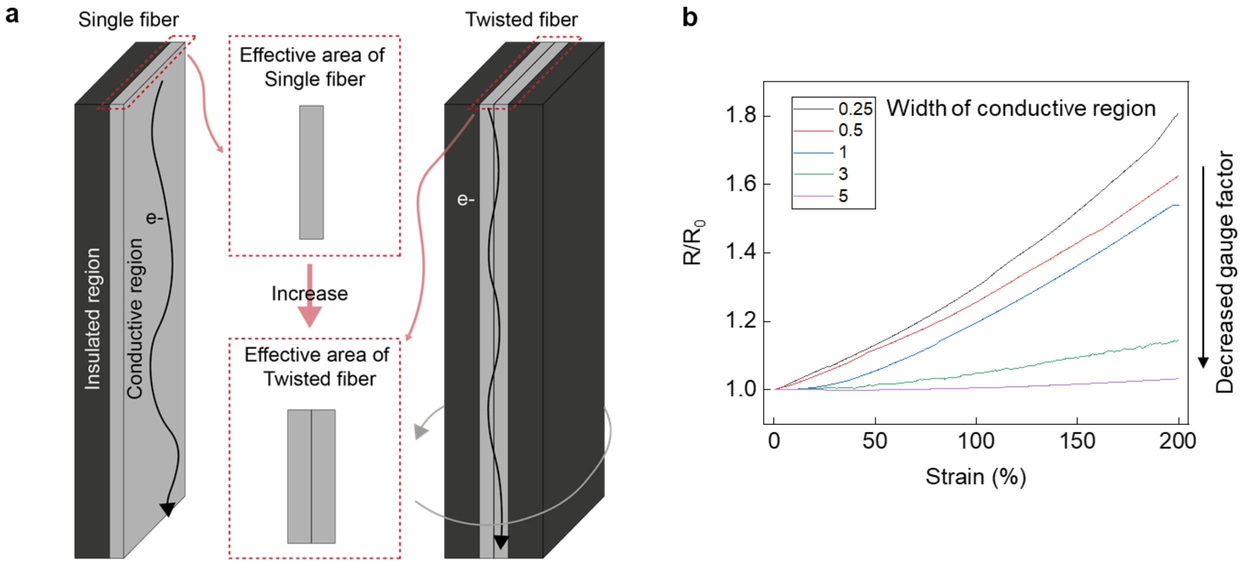

Supplementary Fig. 24| Gauge factor of fiber according to width of conductive area. a, Schematic illustration of increase of conductive region of TSF^{tw} **b,** Resistance variation under strain according to width of conductive region.

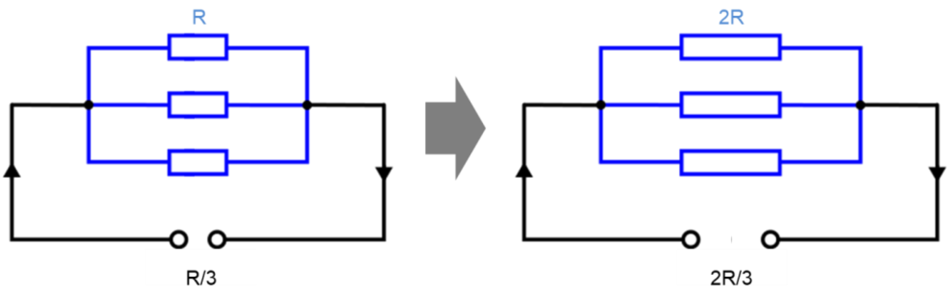

Supplementary Fig. 25| Equivalent circuit of twisted TSF without sintering.

In the absence of twisting or sintering in TSF strands, our TSF operates as a parallel circuit, consequently maintaining an unaffected gauge factor. However, when subjected to twisting or sintering, the effective width of the conductive layer enlarges, resulting in a lower gauge factor compared to a parallel bundle of TSF

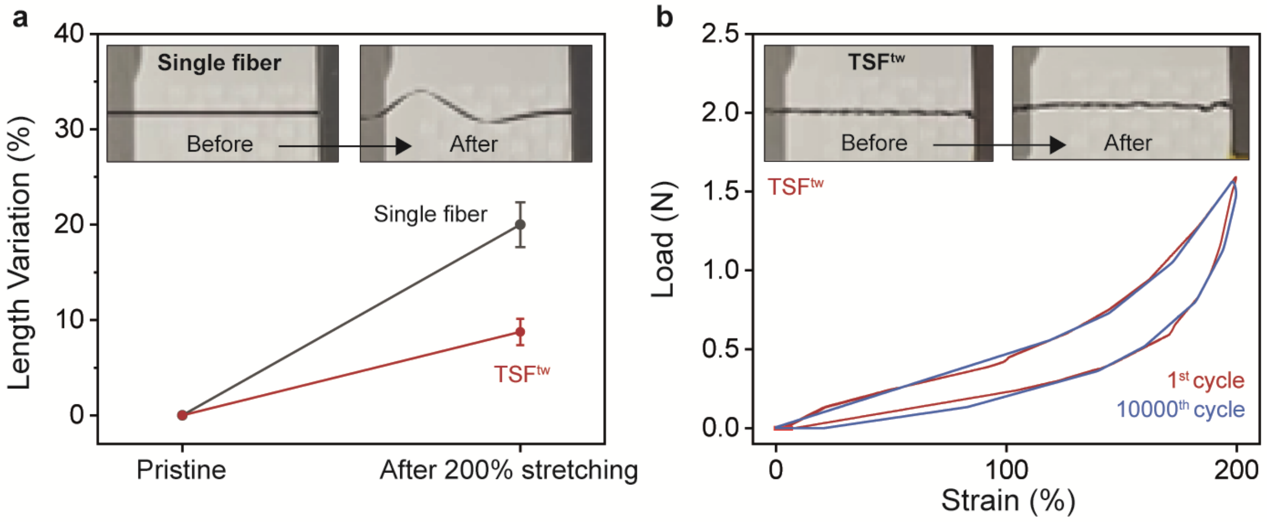

Supplementary Fig. 26| Mechanical properties of single fiber and TSF^{tw}. a, Elongation of TSF and TSF^{tw} after stretching. **b,** Load and strain curve of TSF^{tw} after 10,000 cycles of 200% stretching

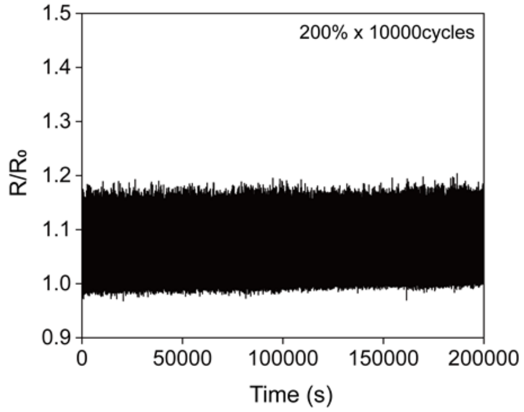

Supplementary Fig. 27| Relative resistance of TSF^{tw}s under applications of 200% stretching.

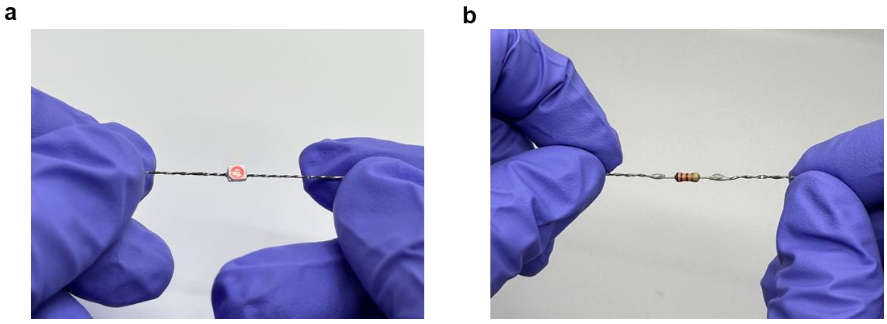

Supplementary Fig. 28| Photograph of electronic integration with TSF^{tw}. a, connection with micro LED. b, integration with 1.2kΩ resistor.

The high toughness and mechanical stability of TSF^{tw} enable robust integration with conventional chips using silver paste.

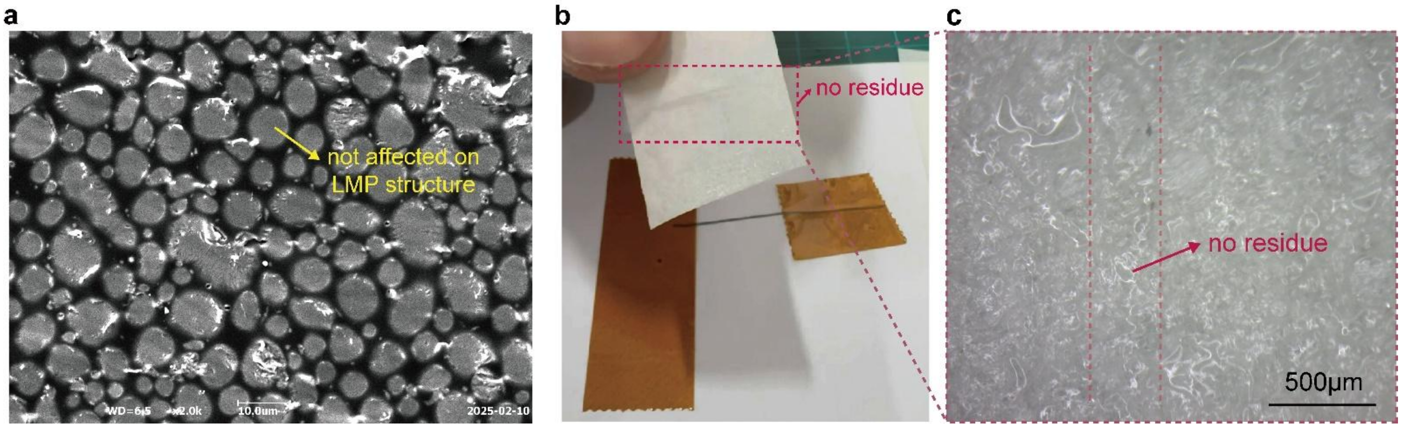

Supplementary Fig. 29| Leakage test by peeling off with Scotch tape. a. SEM image of the fiber after peeling off with Scotch tape. **b.** Photograph of fiber after peel-off test with Scotch tape. **c.** Optical image of scotch tape after peeling off fiber.

Due to the enhanced mechanical stability, there is no rupture of LMP after peeling off with Scotch tape, resulting in no notable residue of LM on the Scotch tape.

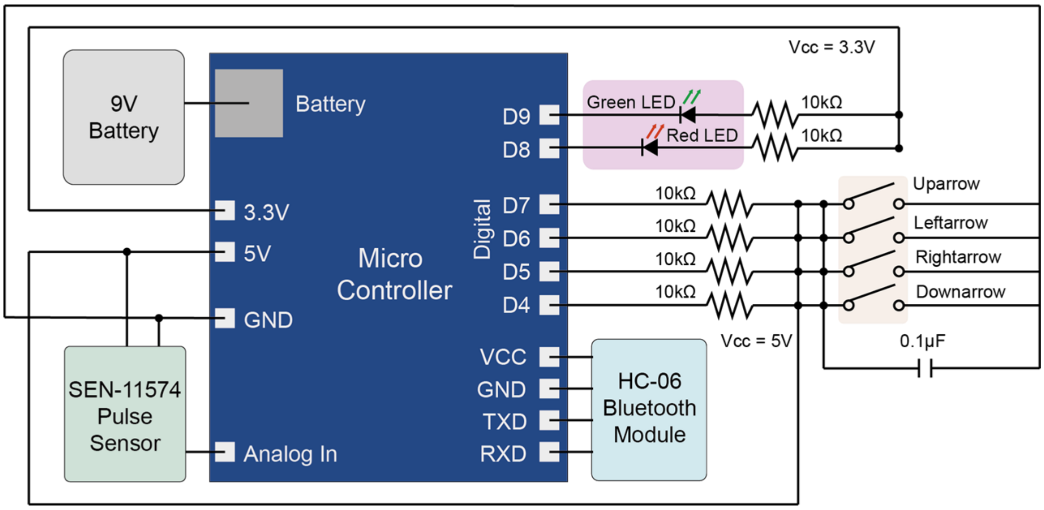

Supplementary Fig. 30| Schematic circuit diagram of TSF^{tw}-based intelligent digital apparel.

To impart various functionalities into digital apparel, we developed an electrical circuit with a microcontroller, interconnected through the TSF^{tw}.

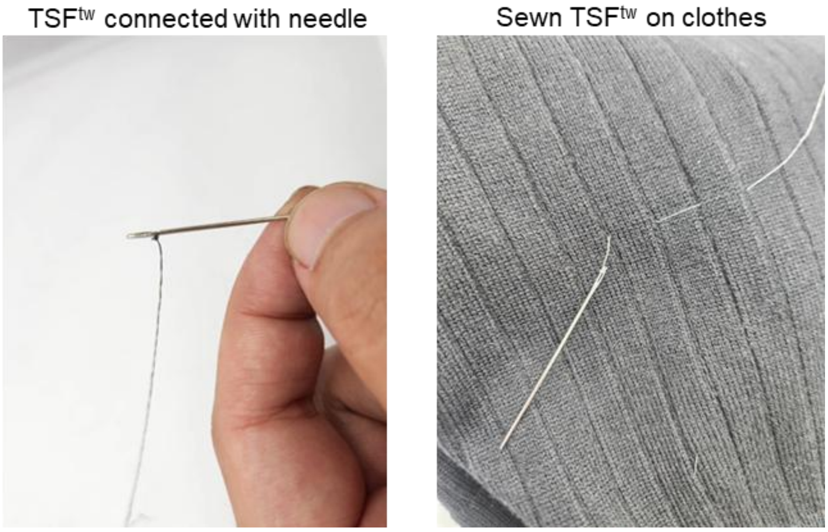

Supplementary Fig. 31| Threading of TSF^{tw} with a needle.

Due to the thin and tough nature of our TSF^{tw}, it can be sutured onto clothing using a standard needle.

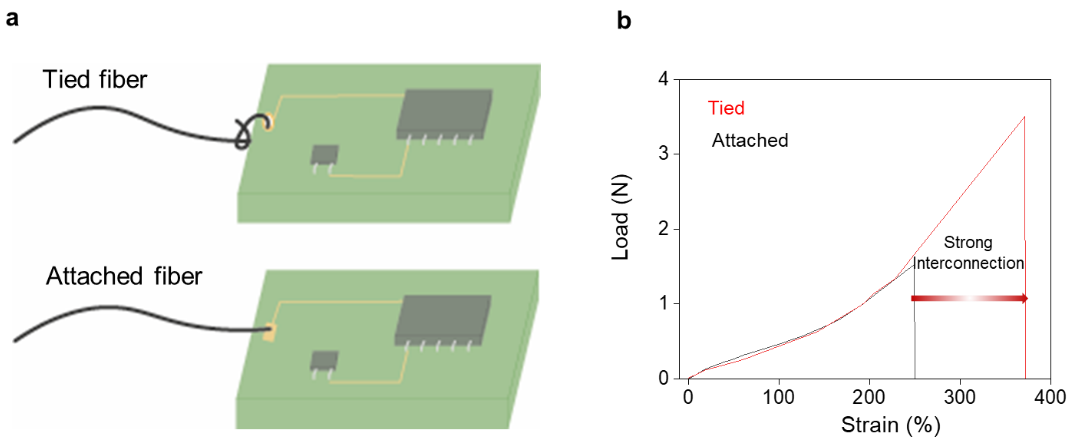

Supplementary Fig. 32| Connection of TSF^{tw} with PCB. a, Schematic illustration of type of connection between fiber and PCB. **b**, Load and strain curve according to connection type.

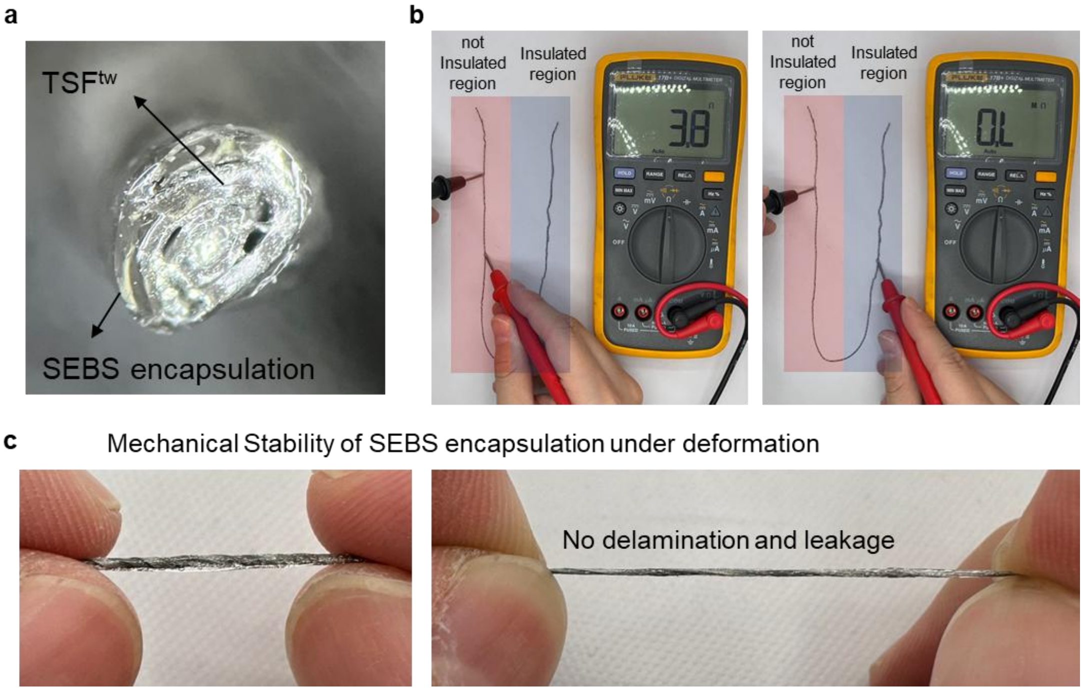

Supplementary Fig. 33| SEBS coating on TSF^{tw}. **a**, Cross-sectional OM image for SEBS-coated TSF^{tw}. **b**, Photograph of TSF^{tw} without and with SEBS coating. **c**, Photograph of stretched SEBS-coated TSF^{tw}.

To prevent unwanted electrical contact and LM leakage during usage, a thin layer of SEBS is applied to TSF^{tw} using a dip-coating method. The use of the non-polar solvent cyclohexane in the SEBS solution ensures that TPU remains undamaged during the conformal coating process. Once coated, SEBS exhibits better stretchability compared to TPU, allowing for stretchable operations without delamination.

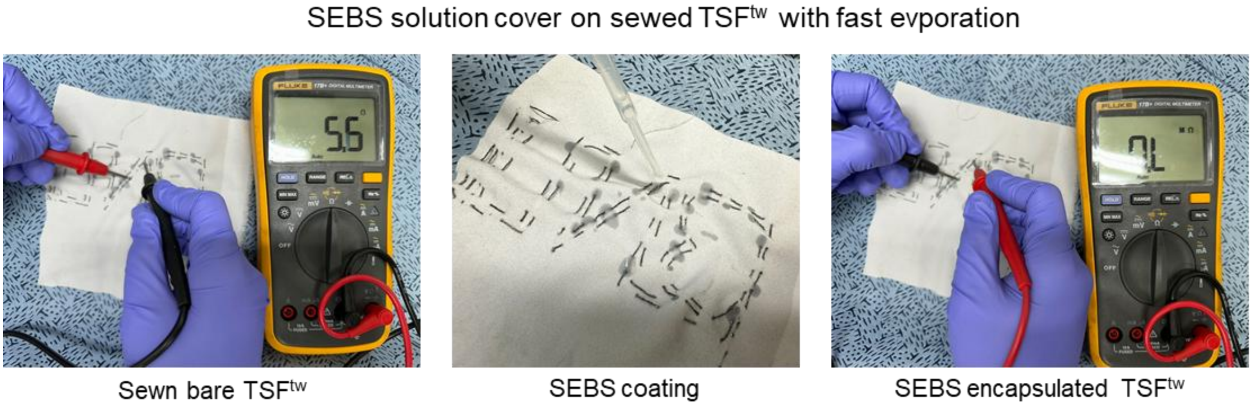

Supplementary Fig. 34| Encapsulation of TSF^{tw} with SEBS for insulation and mechanical durability.

Applying a coating of SEBS onto the sewn TSF^{tw} after its connection with electronic chips provides electrical insulation and increased mechanical durability, effectively preventing any electrical shorting or possible leakage. This is made possible through the use of cyclohexane, a non-polar and highly volatile solvent present in the SEBS solution, enabling a rapid and damage-free coating process.

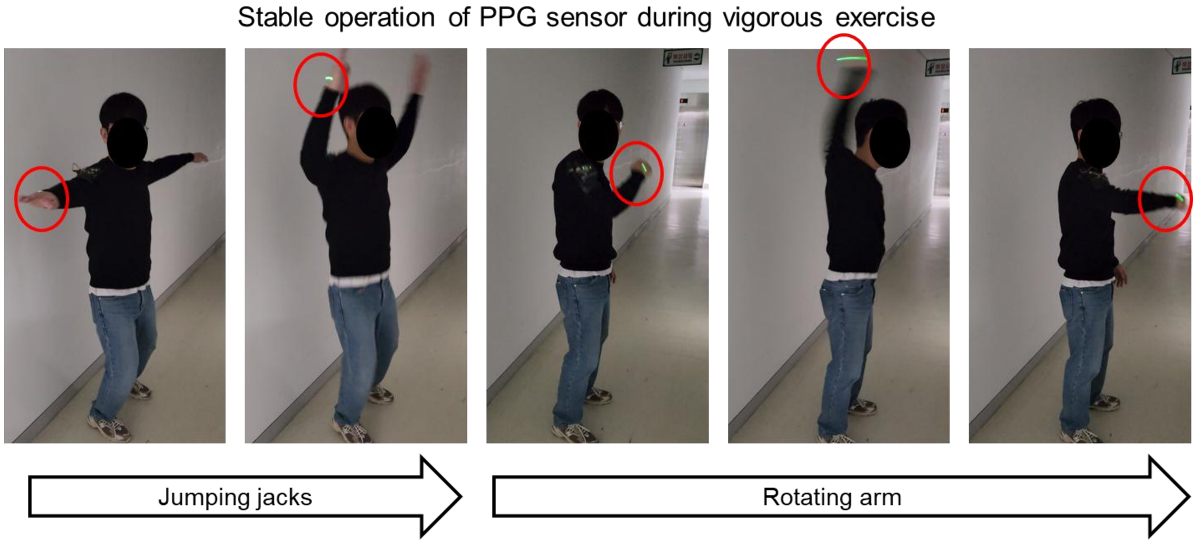

Supplementary Fig. 35| Stable operation of TSF^{tw} under harsh mechanical deformation.

The high toughness and low gauge factor of TSF^{tw} enable stable operation during vigorous body movements, as demonstrated in Supplementary Video 2.

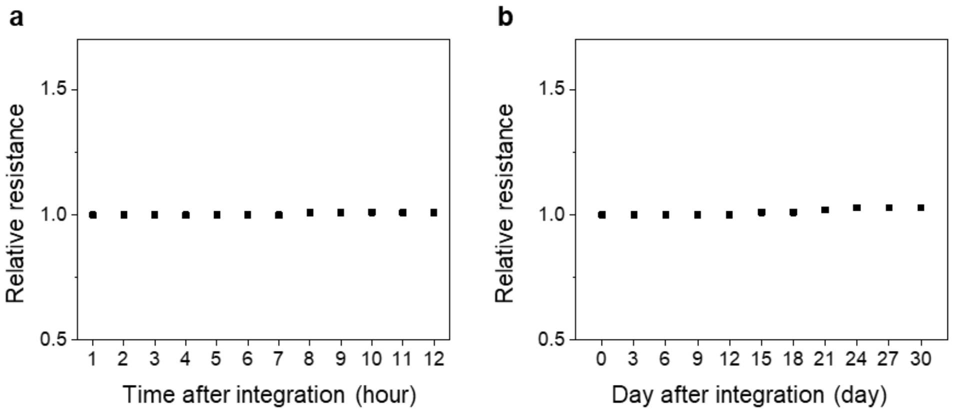

Supplementary Fig. 36| Stable electrical property of TSF^{tw} a, Relative resistance during daily usage. **b**, Relative resistance for long time.

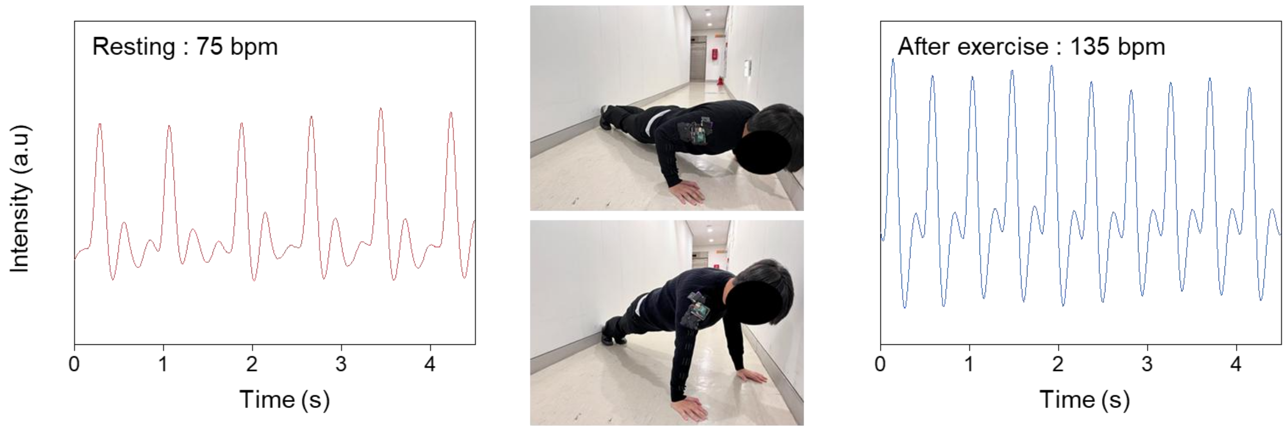

Supplementary Fig. 37| PPG sensor before and after vigorous exercise.

The integrated PPG sensor in IDA can continuously monitor real-time variations in heart activity.

```
function keyboard_ble()
% Create the main GUI figure
    hc06 = bluetooth("BLE1"); % 기본 Pin 1234
% hc06 = bluetooth("HC-06");

fig = figure('Name', 'Object Movement', 'KeyPressFcn', @keyPressCallback);
write(hc06,0)

% Set the axis limits to define the allowable movement area
axisLimits = [0, 100, 0, 100];
axis(axisLimits);

% Hide x and y tick labels
set(gca, 'xtick', [], 'ytick', []);

% Initialize object position and handle
objectPosition = [2.9, 2.9];
objectSize = 5;
objectHandle = rectangle('Position', [objectPosition - objectSize/2, objectSize, objectSize], 'Curvature', [1, 1], 'FaceColor', [0.5 0.7 1],'LineStyle','none');

%       objectHandle = rectangle('Position', [objectPosition, 2, 2], 'Curvature', [1, 1], 'FaceColor', [0.5 0.7 1],'LineStyle','none');

% Define step size for object movement
stepSize = 5;

% Define goal position
goalPosition = [90, 90];
goalSize = 10;
goalHandle = rectangle('Position', [goalPosition, goalSize, goalSize], 'FaceColor',[0.4660 0.6740 0.1880],'LineStyle','none');

% Define uniform obstacle size
obstacleSize = 10;

% Define multiple obstacle positions
obstaclePositions = [0,80;
    10,10;
    10,20;
```

```
        10,40;
        10,60;
        10,80;
        20,0;
        20,20;
        20,30;
        20,50;
        30,10;
        30,20;
        30,50;
        30,70;
        30,80;
        30,90;
        40,40;
        40,70;
        50,10;
        50,20;
        50,60;
        50,80;
        60,30;
        60,40;
        60,50;
        60,70;
        60,80;
        70,0;
        70,10;
        70,30;
        70,90;
        80,50;
        80,60;
        80,80;
        90,10;
        90,20;
        90,40;
        ];

numObstacles = size(obstaclePositions, 1);
obstacleHandles = gobjects(numObstacles, 1);

for i = 1:numObstacles
```

```
    obstacleHandles(i) = rectangle('Position', [obstaclePositions(i, 1), obstaclePositions(i, 2), obstacleSize, obstacleSize], 'Curvature', [0, 0], 'FaceColor', [0.8500 0.3250 0.0980],'LineStyle','none');
end

configureCallback(hc06, "byte", 4, @newData);

    function newData(src, evt)
        src.UserData = read(src,src.BytesAvailableFcnCount);

        newObjectPosition = objectPosition;
        if src.UserData(4) == 0
            newObjectPosition(2) = min(objectPosition(2) + stepSize, axisLimits(4) - objectSize/2);
        end
        if src.UserData(3) == 0
            newObjectPosition(2) = max(objectPosition(2) - stepSize, axisLimits(3)+ objectSize/2);
        end
        if src.UserData(2) == 0
            newObjectPosition(1) = max(objectPosition(1) - stepSize, axisLimits(1)+ objectSize/2);
        end
        if src.UserData(1) == 0
            newObjectPosition(1) = min(objectPosition(1) + stepSize, axisLimits(2) - objectSize/2);
        end

        hasCollision = false;
        for i = 1:numObstacles
            if checkCollision(newObjectPosition, obstaclePositions(i, :), obstacleSize)
                hasCollision = true;
                write(hc06,2)
                break;
            end
        end

        if ~hasCollision
            objectPosition = newObjectPosition;
            updateObjectPosition();
            % Check if the object has reached the goal position

            if isAtGoal(objectPosition, goalPosition, objectSize)
                disp('Goal reached!');
                write(hc06,1)
```

```
                % You can add additional code here to handle the goal reached event
            else
                write(hc06,0)
            end
        end

    end

    function keyPressCallback(~, event)
        % Get the pressed key
        keyPressed = event.Key;

        % Update object position based on the pressed key
        newObjectPosition = objectPosition;
        switch keyPressed
            case 'uparrow'
                newObjectPosition(2) = min(objectPosition(2) + stepSize, axisLimits(4) - objectSize/2);
            case 'downarrow'
                newObjectPosition(2) = max(objectPosition(2) - stepSize, axisLimits(3)+ objectSize/2);
            case 'leftarrow'
                newObjectPosition(1) = max(objectPosition(1) - stepSize, axisLimits(1)+ objectSize/2);
            case 'rightarrow'
                newObjectPosition(1) = min(objectPosition(1) + stepSize, axisLimits(2) - objectSize/2);
        end

%                   % Check for collision with obstacles using an if statement
%                   hasCollision = false;
%                   for i = 1:numObstacles
%                       if checkCollision(newObjectPosition, obstaclePositions(i, :), obstacleSize)
%                           hasCollision = true;
%           %                   1
%                           write(hc06,1)
%                           break;
%                       end
%                   end
%
%                   if ~hasCollision
%
%                       write(hc06,0)
%                       % 0
```

```
                    objectPosition = newObjectPosition;
                    updateObjectPosition();
%
%                     % Check if the object has reached the goal position
%                     if isAtGoal(objectPosition, goalPosition, objectSize)
%                         disp('Goal reached!');
%                         %                   2
%                         write(hc06,2)
%                         % You can add additional code here to handle the goal reached event
%                     end
%                 end
    end

    function collision = checkCollision(positionA, positionB, sizeB)
        % Check if positionA collides with the area of positionB
        collision = (positionA(2) >= positionB(2)) && (positionA(2) <= positionB(2) + sizeB) && ...
            (positionA(1) >= positionB(1)) && (positionA(1) <= positionB(1) + sizeB) ;
        %         && ...
        %             (positionA(1) + objectSize >= positionB(1)) && (positionA(1) + objectSize <=
positionB(1) + sizeB);
    end

    function updateObjectPosition()
        % Update object position in the GUI
        set(objectHandle, 'Position', [objectPosition - objectSize/2, objectSize, objectSize]);
    end

    function atGoal = isAtGoal(currentPosition, goalPosition, objectSize)
        % Check if the object is at the goal position
        atGoal = all(currentPosition >= goalPosition) && all(currentPosition <= goalPosition +
goalSize);
    end

end
```
Supplementary Fig. 38| Bluetooth code for IDA.

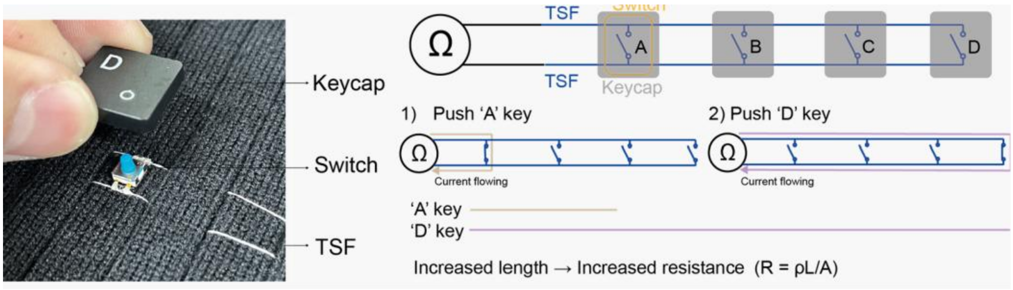

Supplementary Fig. 39| Working principle of clothes-integrated keyboard.

To determine the pressed key, we designed a method based on the resistance variation in accordance with the length of the conductive pathway. Two TSF^{tw} strands are linked via a commercialized switch. When a button is pressed, the switch connects these two TSF^{tw} strands, altering the conductive pathway. As the conductive pathway increases corresponding to the alphabet, we are able to determine the pressed button by measuring the resulting resistance.

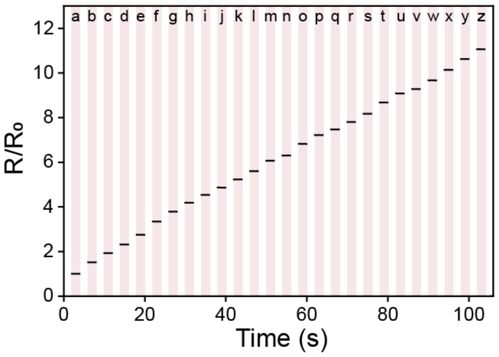

Supplementary Fig. 40| Relative resistance of the keyboard for typing each letter.

When each letter on the keyboard was pressed, the resistance varied according to the length of the fiber electrodes, allowing the keyboard to function.

References

1 Dong, C. Q. *et al.* High-efficiency super-elastic liquid metal based triboelectric fibers and textiles. *Nature Communications* **11** (2020). https://doi.org:10.1038/s41467-020-17345-8

2 Zheng, L. J. *et al.* Conductance-stable liquid metal sheath-core microfibers for stretchy smart fabrics and self-powered sensing. *Science Advances* **7** (2021). https://doi.org:10.1126/sciadv.abg4041

3 Lai, Y. C. *et al.* Elastic Multifunctional Liquid-Metal Fibers for Harvesting Mechanical and Electromagnetic Energy and as Self-Powered Sensors. *Advanced Energy Materials* **11** (2021). https://doi.org:10.1002/aenm.202100411

4 Xiong, Y. *et al.* A multifunctional hollow TPU fiber filled with liquid metal exhibiting fast electrothermal deformation and recovery. *Soft Matter* **17**, 10016-10024 (2021). https://doi.org:10.1039/d1sm01189h

5 Lin, R. Z. *et al.* Digitally-embroidered liquid metal electronic textiles for wearable wireless systems. *Nature Communications* **13** (2022). https://doi.org:10.1038/s41467-022-29859-4

6 Long, L. F. *et al.* Interfacial Electrochemical Polymerization for Spinning Liquid Metals into Core-Shell Wires. *Acs Applied Materials & Interfaces* **14**, 18690-18696 (2022). https://doi.org:10.1021/acsami.2c02247

7 Chen, M. X. *et al.* Self-powered multifunctional sensing based on super-elastic fibers by soluble-core thermal drawing. *Nature Communications* **12** (2021). https://doi.org:10.1038/s41467-021-21729-9

8 Sun, F. *et al.* Stretchable conductive fibers of ultrahigh tensile strain and stable conductance enabled by a worm-shaped graphene microlayer. *Nano Lett.* **19**, 6592-6599 (2019).

9 Zhang, B. *et al.* Stretchable conductive fibers based on a cracking control strategy for wearable electronics. *Adv. Funct. Mater.* **28**, 1801683 (2018).

10 Zhao, Y. *et al.* A moss-inspired electroless gold-coating strategy toward stretchable fiber conductors by dry spinning. *Advanced Electronic Materials* **5**, 1800462 (2019).

11 Matsuhisa, N. *et al.* Printable elastic conductors with a high conductivity for electronic textile applications. *Nature communications* **6**, 7461 (2015).

12 Chen, G. *et al.* Superelastic EGaIn composite fibers sustaining 500% tensile strain with superior electrical conductivity for wearable electronics. *ACS Applied Materials & Interfaces* **12**, 6112-6118 (2020).

13 Ma, Z. *et al.* Stretchable and conductive fibers fabricated by a continuous method for wearable devices. *Cell Reports Physical Science* **4** (2023).

14 Jung, J., Jeong, S. H., Hjort, K. & Ryu, S. Investigation of thermal conductivity for liquid metal composites using the micromechanics-based mean-field homogenization theory. *Soft Matter* **16**, 5840-5847 (2020). https://doi.org:10.1039/d0sm00279h
