# Supplementary material for: Meter-scale heterostructure printing for high-toughness fiber electrodes in intelligent digital apparel
Source: Nat Commun. 2025 May 9;16:4320. doi: 10.1038/s41467-025-59703-4 (PMC12064786; doi:10.1038/s41467-025-59703-4)
Supplement: Supplementary file 2 — Description of Additional Supplementary Files [file 41467_2025_59703_MOESM2_ESM.docx]

Description of Additional Supplementary Files

**File Name:** Supplementary Data 1

**Description:** Demonstration of meter-scale stretchable fiber.

**File Name:** Supplementary Data 2

**Description:** Achieving electrical conductivity during detachment.

**File Name:** Supplementary Data 3

**Description:** Functionality of digital apparel during physical activity.

**File Name:** Supplementary Data 4

**Description:** Human–computer interaction through digital apparel.
